# Correction: Young people’s data governance preferences for their mental health data: MindKind Study findings from India, South Africa, and the United Kingdom

**DOI:** 10.1371/journal.pone.0336686

**Published:** 2025-11-11

**Authors:** Solveig K. Sieberts, Carly Marten, Emily Bampton, Elin A. Björling, Anne-Marie Burn, Emma Grace Carey, Sonia Carlson, Blossom Fernandes, Jasmine Kalha, Simthembile Lindani, Hedwick Masomera, Augustina Mensa-Kwao, Lakshmi Neelakantan, Lisa Pasquale, Swetha Ranganathan, Erin Joy Scanlan, Himani Shah, Refiloe Sibisi, Sushmita Sumant, Christine Suver, Yanga Thungana, Meghasyam Tummalacherla, Jennifer Velloza, Pamela Y. Collins, Mina Fazel, Tamsin Ford, Melvyn Freeman, Soumitra Pathare, Zukiswa Zingela, The MindKind Consortium, Megan Doerr

Augustina Mensa-Kwao is not included in the author byline. Augustina Mensa-Kwao should be listed as the twelfth author and affiliated with Department of Global Health, University of Washington, Seattle, Washington, United States of America, Department of Psychiatry and Behavioral Sciences, University of Washington, Seattle, Washington, United States of America. The contributions of this author are as follows: Conceptualization, Methodology, Writing – review & editing.
