# Supplementary material for: Young people’s data governance preferences for their mental health data: MindKind Study findings from India, South Africa, and the United Kingdom
Source: PLoS One. 2023 Apr 19;18(4):e0279857. doi: 10.1371/journal.pone.0279857 (PMC10115253; doi:10.1371/journal.pone.0279857)
Supplement: S1 File — (PDF) [file pone.0279857.s001.pdf]

Supporting Information for

Young people's data governance preferences for their mental health data: MindKind Study findings from India, South Africa, and the United Kingdom

Table of Contents

Figures **2**
S1 Fig. Age dependent preference for data access control for India (IN), South Africa (SA) and UK. 2
S2 Fig. Deliberative outputs among Indian participants only. 3
S4 Fig. Deliberative outputs among UK participants only. 4
Tables **5**
S1 Table. Participant characteristics by country. 5
S2 Table. Multinomial regression model for “How should researchers be able to access your data.” 6
S3 Table. Multinomial regression model for “Who controls the data.” 7
S4 Table. Logistic regression model for enrollment by consent model (all sites). 8
S5 Table. Logistic regression model for enrollment by consent model (by country). 9
S6 Table. Contrasts for Democracy vs Defined Terms (Group C & D vs Group B) and server data access vs data download (Group D vs Groups B & C). 10
S7 Table. Results of democratic voting. 11
Supporting Methods **12**
Democratic choice voting 12
Facilitation Guide 13
Supporting Results **16**
Deliberative Outputs: Argument Mapping 16

Figures

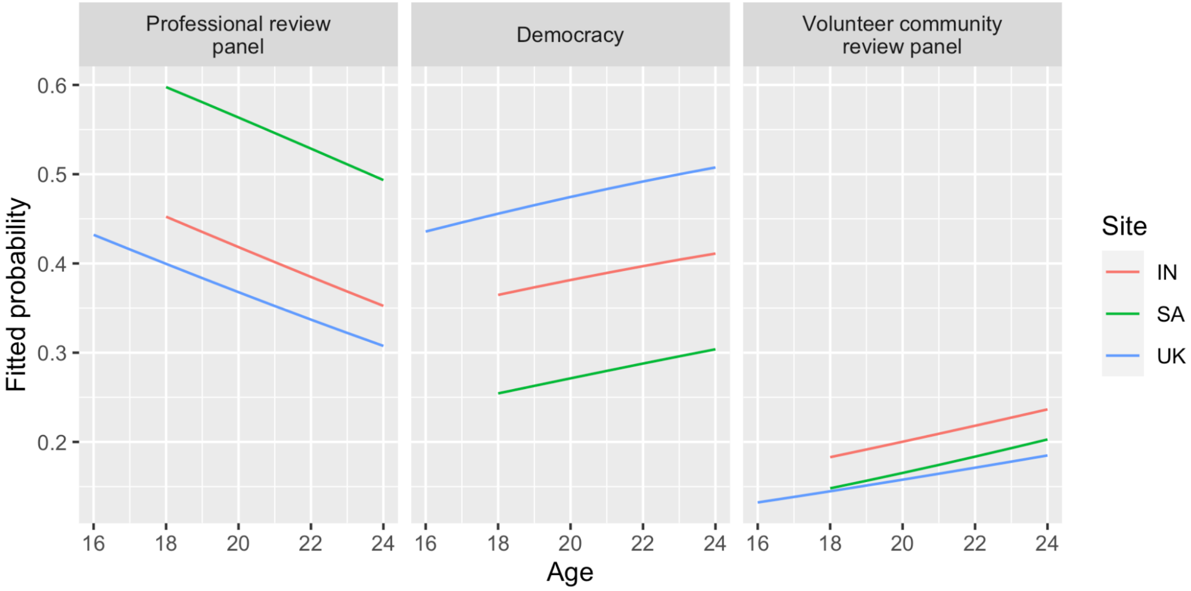

S1 Fig. Age dependent preference for data access control for India (IN), South Africa (SA) and UK.

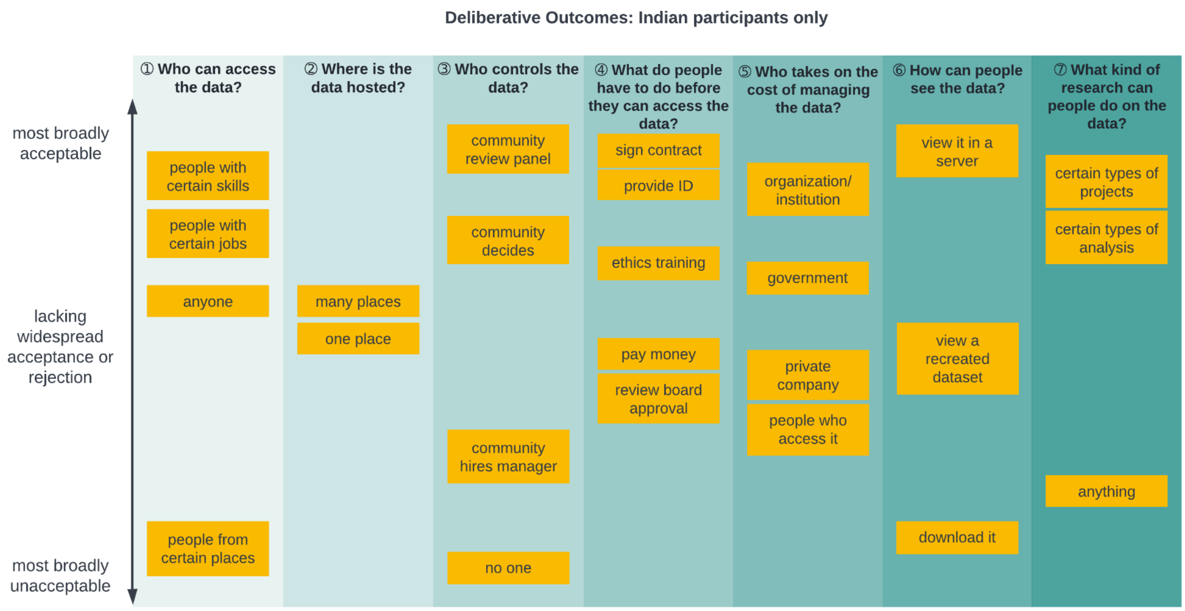

S2 Fig. Deliberative outputs among Indian participants only.

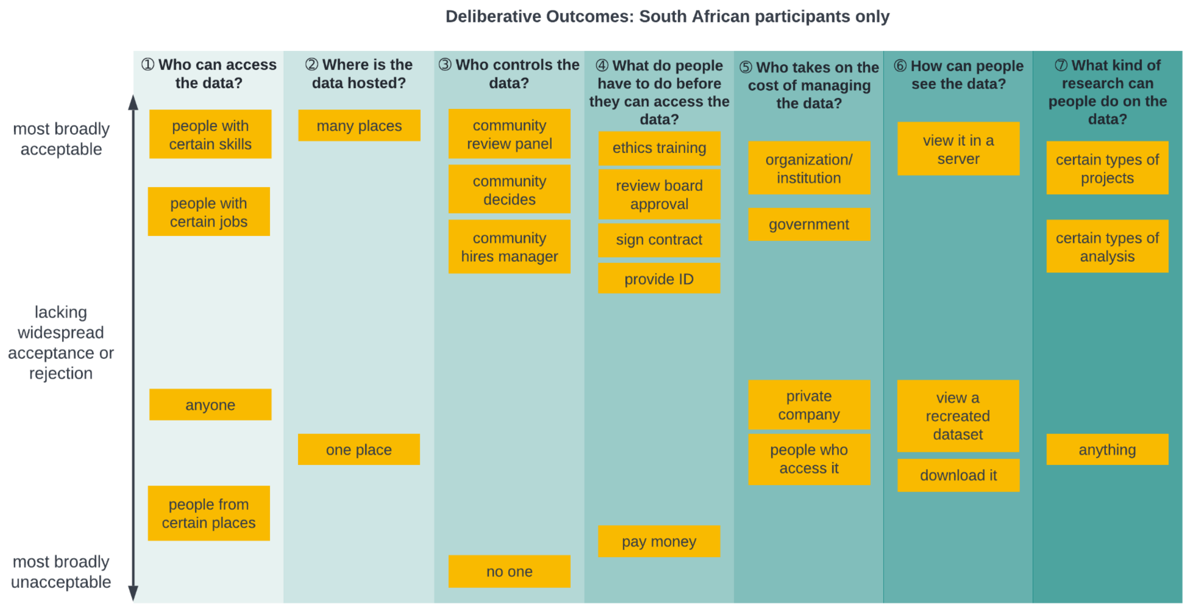

S3 Fig. Deliberative outputs among South African participants only.

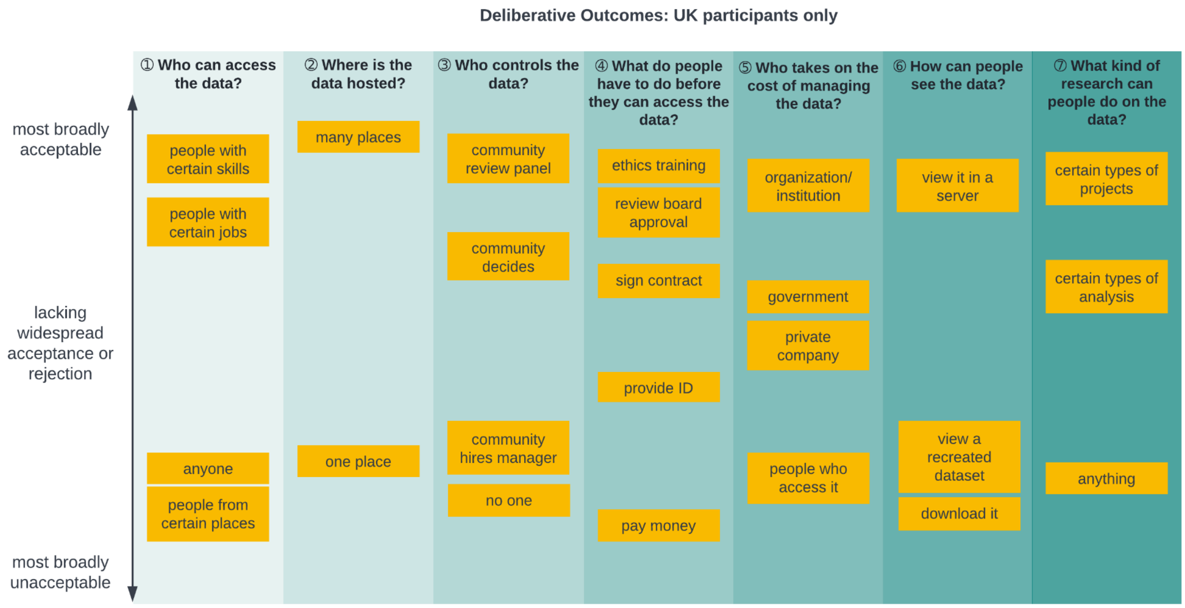

S4 Fig. Deliberative outputs among UK participants only.

Tables

S1 Table. Participant characteristics by country.

	Consented			Unconsented		
**Characteristic**	**India**, N = 1,034[1]	**South Africa**, N = 932[1]	**UK**, N = 1,609[1]	**India**, N = 1,409[1]	**South Africa**, N = 710[1]	**UK**, N = 1,045[1]
**Age**						
16	0 (0%)	0 (0%)	349 (22%)	0 (0%)	0 (0%)	256 (24%)
17	0 (0%)	0 (0%)	424 (26%)	0 (0%)	0 (0%)	297 (28%)
18	178 (17%)	87 (9.3%)	129 (8.0%)	218 (15%)	61 (8.6%)	91 (8.7%)
19	163 (16%)	117 (13%)	135 (8.4%)	210 (15%)	87 (12%)	80 (7.7%)
20	158 (15%)	146 (16%)	142 (8.8%)	239 (17%)	102 (14%)	78 (7.5%)
21	166 (16%)	179 (19%)	114 (7.1%)	203 (14%)	116 (16%)	65 (6.2%)
22	134 (13%)	153 (16%)	106 (6.6%)	212 (15%)	113 (16%)	62 (5.9%)
23	125 (12%)	134 (14%)	108 (6.7%)	183 (13%)	119 (17%)	65 (6.2%)
24	110 (11%)	116 (12%)	102 (6.3%)	144 (10%)	112 (16%)	51 (4.9%)
**Gender**						
Woman	897 (87%)	737 (79%)	1,031 (64%)	1,270 (90%)	578 (81%)	652 (62%)
Man	103 (10.0%)	162 (17%)	247 (15%)	102 (7.2%)	104 (15%)	219 (21%)
Transgender/Nonbinary/Multiple/Other	21 (2.0%)	23 (2.5%)	296 (18%)	16 (1.1%)	18 (2.5%)	153 (15%)
Prefer not to say	13 (1.3%)	10 (1.1%)	35 (2.2%)	21 (1.5%)	10 (1.4%)	21 (2.0%)
**Lived Experience**						
Lived Experience	908 (88%)	623 (67%)	1,469 (91%)	1,188 (84%)	446 (63%)	921 (88%)
No Lived Experience	126 (12%)	309 (33%)	140 (8.7%)	221 (16%)	264 (37%)	124 (12%)
**How did you hear about the study?**						
Health clinic contact	0 (0%)	8 (0.9%)	0 (0%)	3 (0.2%)	4 (0.6%)	0 (0%)
Other	35 (3.4%)	17 (1.8%)	11 (0.7%)	33 (2.3%)	24 (3.4%)	9 (0.9%)
Paper flyer	0 (0%)	2 (0.2%)	5 (0.3%)	0 (0%)	0 (0%)	2 (0.2%)
Prefer not to say	9 (0.9%)	3 (0.3%)	4 (0.2%)	8 (0.6%)	2 (0.3%)	1 (<0.1%)
Referred by a friend	84 (8.1%)	79 (8.5%)	30 (1.9%)	88 (6.2%)	48 (6.8%)	9 (0.9%)
School or University contact	72 (7.0%)	204 (22%)	106 (6.6%)	72 (5.1%)	109 (15%)	54 (5.2%)
Social media advertisement	828 (80%)	606 (65%)	1,445 (90%)	1,198 (85%)	511 (72%)	965 (92%)
Web browser search	6 (0.6%)	13 (1.4%)	8 (0.5%)	7 (0.5%)	12 (1.7%)	5 (0.5%)

[1] n (%)

S2 Table. Multinomial regression model for "How should researchers be able to access your data."

	Researchers should be allowed to download a copy of my data.*			Researchers should have to ask a data steward to run an analysis of my data and return the results to them.*		
	Coefficient	SE	p-value	Coefficient	SE	p-value
(Intercept)	-1.309	0.779	0.093	-2.445	0.936	0.009
Age	0.029	0.036	0.42	0.057	0.043	0.19
Gender (Woman)	-	-	-	-	-	-
Gender (Man)	-0.202	0.572	0.724	0.545	0.472	0.248
Gender (Transgender/ Nonbinary/ Multiple/Other)	-0.463	0.452	0.306	0.489	0.411	0.234
Gender (Prefer not to say)	0.203	0.286	0.478	0.727	0.339	0.032
Country (IN)	-	-	-	-	-	-
Country (SA)	0.29	0.22	0.188	0.472	0.245	0.054
Country (UK)	0.303	0.203	0.136	-0.066	0.243	0.784
Lived Experience (True)	0.01	0.22	0.965	0.412	0.277	0.138
	Residual Deviance: 1789.764					
	AIC: 1821.764					

* Relative to "Researchers should only be allowed to see my data in a secure server, but cannot download my data."

S3 Table. Multinomial regression model for "Who controls the data."

	Democracy*			Volunteer Community Review Panel*		
	Coefficient	SE	p-value	Coefficient	SE	p-value
(Intercept)	-1.494	0.75	0.046	-2.592	0.979	0.008
Age	0.068	0.035	0.05	0.088	0.045	0.05
Gender (Woman)	-	-	-	-	-	-
Gender (Man)	0.18	0.439	0.681	-0.378	0.748	0.613
Gender (Transgender/ Nonbinary/ Multiple/Other)	-0.153	0.362	0.671	-0.515	0.594	0.386
Gender (Prefer not to say)	-0.286	0.262	0.275	-0.314	0.381	0.409
Country (IN)	-	-	-	-	-	-
Country (SA)	-0.61	0.213	0.004	-0.504	0.255	0.048
Country (UK)	0.29	0.194	0.136	-0.159	0.248	0.522
Lived Experience (True)	0.176	0.22	0.425	0.035	0.268	0.896
	Residual Deviance: 1828.731					
	AIC: 1860.731					

* Relative to Professional Review Panel.

S4 Table. Logistic regression model for enrollment by consent model (all sites).

	Univariate Model				Multivariate Model			
**Characteristic**	**N**	**OR[1]**	**95% CI[1]**	**p-value**	**N**	**OR[1]**	**95% CI[1]**	**p-value**
(Intercept)	5,057	1.05	0.95, 1.15	0.4	5,057	0.57	0.33, 1.00	0.050
Consent Model								
B: Researcher Norms	1,667	—	—		1,667	—	—	
C: Democracy/Download	1,695	1.11	0.97, 1.27	0.13	1,695	1.12	0.97, 1.28	0.11
D: Democracy/Server	1,695	1.11	0.97, 1.27	0.13	1,695	1.12	0.98, 1.28	0.11
Age					5,057	1.00	0.97, 1.02	0.9
Gender								
Woman					3,870	—	—	
Man					703	1.05	0.89, 1.24	0.6
Transgender/ Nonbinary/ Multiple/Other					396	1.33	1.06, 1.68	0.013
Prefer not to say					88	0.98	0.64, 1.51	>0.9
Country								
IN					1,845	—	—	
SA					1,222	1.88	1.62, 2.19	<0.001
UK					1,990	1.97	1.71, 2.28	<0.001
Lived Experience								
FALSE					884	—	—	
TRUE					4,173	1.26	1.08, 1.47	0.003

[1] OR = Odds Ratio, CI = Confidence Interval

S5 Table. Logistic regression model for enrollment by consent model (by country).

	India				South Africa				UK			
**Characteristic**	**N**	**OR**[1]	**95% CI**[1]	**p-value**	**N**	**OR**[1]	**95% CI**[1]	**p-value**	**N**	**OR**[1]	**95% CI**[1]	**p-value**
(Intercept)	1,845	0.71	0.60, 0.84	<0.001	1,222	1.28	1.05, 1.55	0.014	1,990	1.31	1.12, 1.53	<0.001
Consent Model												
B: Researcher Norms	599	—	—		412	—	—		656	—	—	
C: Democracy/Download	614	1.11	0.88, 1.39	0.4	425	0.98	0.74, 1.29	0.9	656	1.22	0.98, 1.53	0.072
D: Democracy/Server	632	0.97	0.77, 1.22	0.8	385	1.11	0.84, 1.48	0.5	678	1.29	1.04, 1.61	0.023

[1] OR = Odds Ratio, CI = Confidence Interval

S6 Table. Contrasts for Democracy vs Defined Terms (Group C & D vs Group B) and server data access vs data download (Group D vs Groups B & C).

	Democracy vs. Pre-defined Terms			Server vs. Download		
	OR	95% CI[1]	*p*-value[2]	OR	95% CI[1]	*p*-value[2]
All Combined	1.11	0.99, 1.25	0.081	1.05	0.94, 1.18	0.398
India	1.04	0.85, 1.26	0.713	0.92	0.76, 1.12	0.403
South Africa	1.04	0.82, 1.33	0.725	1.13	0.88, 1.44	0.342
UK	1.26	1.04, 1.52	0.018	1.17	0.96, 1.41	0.114

[1] CI = Confidence Interval
[2] Un-adjusted *p*-value

S7 Table. Results of democratic voting.

Question	**India**, N = 628[1]	**South Africa**, N = 529[1]	**UK**, N = 1,018[1]
**Can my data be used by researchers to make a profit?**			
Yes, my data can be used by researchers to make a profit.	187 (30%)	170 (32%)	109 (11%)
No, my data can NOT be used by researchers to make a profit.	353 (56%)	307 (58%)	610 (60%)
I don't care if my data is used by researchers to make a profit..	88 (14%)	52 (9.8%)	298 (29%)
**Do people have to pay to use my data?**			
Nobody should have to pay to use my data.	216 (34%)	190 (36%)	369 (36%)
Only commercial companies should have to pay to use my data.	365 (58%)	310 (59%)	468 (46%)
I don't care if people have to pay to use my data.	47 (7.5%)	29 (5.5%)	181 (18%)
**How can my data be used?**			
My data should be available for broad research purposes.	117 (19%)	77 (15%)	213 (21%)
My data should be available for all types of health research.	160 (25%)	172 (33%)	397 (39%)
My data should only be available for mental health research.	320 (51%)	262 (50%)	307 (30%)
I don't care how my data is used.	31 (4.9%)	18 (3.4%)	101 (9.9%)
**How can results be shared with participants?**			
Results should be shared for free with the world.	57 (9.1%)	65 (12%)	98 (9.6%)
Results should be shared in an easy to understand way with participants.	206 (33%)	215 (41%)	265 (26%)
Both are important to me.	352 (56%)	242 (46%)	592 (58%)
I don't care how results are shared with participants.	13 (2.1%)	7 (1.3%)	63 (6.2%)

[1] n (%)

Supporting Methods

Democratic choice voting

Participants randomized to Groups C and D, as well as those that selected the Democracy Choice option for Choice 2 in Group A, were prompted to vote on 4 questions covering (1) whether data could be used for for-profit endeavors, (2) whether researchers have to pay to use the data, (3) the types of research the data can be used for, and (4) how results should be shared. These options were not given to those participants randomized to Group B, nor those that selected Volunteer or Professional Review Panel for Choice 2.

- Can my data be used by researchers to make a profit?
  - Yes, my data can be used by researchers to make a profit.
  - No, my data can NOT be used by researchers to make a profit.
  - I don't care if my data is used by researchers to make a profit.
- Do people have to pay to use my data?
  - Only commercial companies should have to pay to use my data.
  - Nobody should have to pay to use my data.
  - I don't care if people have to pay to use my data.
- How can my data be used?
  - My data should only be available for mental health research.
  - My data should be available for all types of health research.
  - My data should be available for broad research purposes.
  - I don't care how my data is used.
- How can results be shared with participants?
  - Results should be shared for free with the world.
  - Results should be shared in an easy to understand way with participants.
  - Both are important to me.
  - I don't care how results are shared with participants.

Facilitation Guide

I. **Terms of Agreement (Ground Rules the group agrees on)**
   - Listen to and respect each other's perspectives.
   - Keep the information shared confidential.
   - Discussion is being recorded for transcription purposes, but participants may not record, take photo or video, or take screenshots during the conversation.
   - Do not interrupt each other.
   - Participants are welcome to leave to attend to their needs.
   - If participants do not want to verbally share an idea, they may write it in the chat box [if available] or send a message to the facilitator.
   - [Discuss options for "hand raising" or other functionality based on platform.]
   - [Discuss with participants: preferences on video on vs. off, muting.]
   - [Additional ground rule suggestions from participants.]

II. **Icebreaker:** introductions in a line or two followed by an icebreaker activity.

III. **Recap of educational materials**

The facilitator will take the participants through a brief recap of educational materials, that is, the four animal models, their characteristics, and how they are described across the seven 'questions'. Before the recap, facilitator sets expectations for the upcoming poll of their preferred model.

IV. **Things to remember**

Before the discussion begins, the facilitator will emphasize that there are no right or wrong answers and that the purpose of the discussion is to understand what the group thinks and feels about sharing their mental health data. Also, participants will be reminded that they do not have to restrict themselves to the four models; they can make their own by picking features that they like. The facilitator will reiterate this throughout the discussion as required.

V. **Favourite Model Poll: pick your most preferred model**

This is the first question asked to the group. Participants will pick one favourite model; this will be anonymous. An additional 'none of the above' option will be available, which does not confine the participants to just the four models.

When the poll is closed, the second facilitator will note the model that is most picked and also least picked. The discussion will open with the following probes,

> **Fav Model: What do you like the most about it? What do you not like?**
>
> **Least Fav Model: What do you not like about it? Is there something you like?**

VI. **Discussion of each component/feature:**

Each of the 'seven questions' will be discussed. The second facilitator will capture pointers from the discussion on the slide in real time.

1. **Who can access the data**

   Anyone, people with certain jobs, people with certain skills, people from certain places

   Who do you think should have access to young people's mental health data, why?

   Who do you think should not have access to young people's mental health data, why?

   Task as a group: Put the groups of people (i.e. anyone, people with certain jobs) into one of three columns i.e., acceptable/maybe/unacceptable for accessing data. *Through this task, benefits, harms and concerns can be elicited.*

2. **Where is the data hosted**

   One place, many places

   Facilitator gives example of how one/many places of storage would translate in real life settings.

   Where should the data be hosted? Why?

3. **Who controls the data**

   No one, community decides, community review panel, community hires manager *(may need to explain what is meant by 'controls')*

   What are your concerns about who controls the data?

   What are the advantages/disadvantages of each choice?

4. **What do people have to do before they can access the data**

   Ethics training, provide ID, review board approval, sign contract, pay money

   What should be the process people need to follow to access this data? What can be the steps of this process? Why are these necessary?

5. **Who takes on the cost of managing the data**

   People who access it, government, organisation/institution, private company

   What will be the implications of who takes on this cost?

   What are the advantages/disadvantages of who takes on this cost?

   Who should take on/not take on this cost, why?

6. **How can people see the data**

   Download it, view it in a server, view a recreated dataset

How should people be able to see this data? What made you choose your answer?

What are the advantages/disadvantages of the different mediums?

7. **What kind of research can people do on the data**

   Anything, certain types of analysis, certain types of projects

   What kind of research should be allowed with this? Why?

   What should not be allowed, why?

   What are your concerns about this?

VII. **Group's model: preferences from discussion for consensus.**

The second facilitator/note-taker puts a table on the slide with the preferred set of features (based on the output from the above discussion) for the group to see and comment on. The second facilitator also summarizes, giving the group time to think one last time before the discussion closes. Could use the raise hand feature for consensus.

The facilitators will highlight group's agreement/disagreement on the preferences.

VIII. Group's non-negotiables for the model (E.g. "It is unacceptable to us to have a databank hosted in one place.")

The facilitator tries to capture what the group thinks is unacceptable when it comes to their use/access/sharing of their mental health data in terms of a global mental health databank.

The facilitators will highlight group's agreement/disagreement on the unacceptable features.

IX. **Closing Polls:**

a. **What is your most important organizing question from the discussed 7 questions?**
b. **What is your least important organizing question from the discussed 7 questions?**

The participants will answer these two polls before the discussion closes.

X. **Share link to exit survey**

Supporting Results

Deliberative Outputs: Argument Mapping

Table of Contents

1. *Who can access the data?*

- Allowing **anyone** to access the data is a means of ensuring equality.
- **Anyone** is okay as long as it's for a good reason and/or purpose.
- When **people with certain jobs** or **certain skills** handle the data, they know how to use it properly and/or they won't misuse it.
- Just because people have **certain jobs** or **certain skills** doesn't mean they'll do the right thing.
- **People from certain places** is discriminatory.
- **People from certain places** limits the cross-cultural accessibility of research.

2. *Where is the data hosted?*

- When data is stored in **many places** it offers us decentralised power and control.
- The data should be held in **many places** but not *that* many.
- The data should be held in **many places**, but everyone should follow GDPR and/or a similar data protection law.
- Data can't be stored in only **one place** because a fire could burn the data centre down.
- Data can't be stored in only **one place** because it could be hacked or lost.
- When data is stored in **one place** it offers us more privacy and control.
- When data is stored in **one place** it offers ease of management/organisation.

3. *Who controls the data?*

- **No one** is concerning because you don't know what could be done with the data.
- **Community decides** would be the best way to fairly represent the entire community.
- **Community decides** would be the best ideally, but it would involve too many people, making it chaotic and/or time consuming.
- **Community decides** could result in a vocal minority having an outsized say.
- **Community hires manager** could concentrate power unduly in one person, leading to bias or overwork.
- **Community hires manager** would be too burdensome for one person to manage such a big dataset.
- A **community review panel** would be more representative of the community and/or representative of areas of expertise.
- A **community review panel** may be too bureaucratic.
- We would like a hybrid of **community decides** + **community hires manager** or **community decides** + **community review panel**.

4. *What do people have to do before they can access the data?*

- Requiring researchers to **provide ID** would help us hold people accountable.
- Requiring researchers to **provide ID** could help us correctly identify people.
- Requiring researchers to **provide ID** could be discriminatory.
- A **contract** can be forged/one could deny that one signed it.
- A **contract** could state the purpose for wanting to access the data.
- A **contract** offers accountability for misuse.
- Requiring researchers to **pay money** could be discriminatory.
- Requiring researchers to **pay money** helps sustain the databank.
- Requiring researchers to **pay money** demonstrates buy-in that protects against misuse.

- Requiring researchers to **pay money** could be done on a sliding scale.
- Requiring researchers to **pay money** may instil corruption.
- A **review board** could assist in ascertaining researchers' economic backgrounds for a sliding scale option.
- A **review board** should be utilised for sensitive, contentious, or for-profit projects.
- Some people might just click through/fast forward an **ethics training**.
- Some professionals already have a code of ethics and do not need an **ethics training**.

5. Who takes on the cost of managing the data?

- [**People who access it**: lines of reasoning mirrored **pay money** above.]
- A **government** is an obvious funder of the databank because the databank serves the welfare of the people.
- A **government** shouldn't fund the databank because that is taxpayers' money—like a backdoor way of making people pay for it.
- The **government** funding the databank would only be used to further their political agenda.
- Having only one **government** pay for the databank would be unfair, as it is global.
- Not all **governments** would be able to afford or prioritise funding the databank.
- If a **private company** funds the databank, they will have an outsized say in what is done with the data.
- Having a **private company** fund the databank is reasonable if the company is making a product that benefits people with mental illness.
- Having a **private company** fund the databank may benefit the company, but it benefits us in that it helps sustain the databank.
- If a **private company** funds the databank, they may use it to make targeted ads.
- If a **private company** funds the databank, they may leak our information.
- A hybrid of several of these (such as **government** + **organisation/institution** + **private company**) is needed to fund the databank.

6. How can people see the data?

- A **server** is a secure option that lacks the disadvantages of other options.
- A **server** makes it challenging to do statistical analyses.
- A **recreated dataset** may not be granular enough to capture our diversity.
- A **recreated dataset** is needed for its privacy because this is sensitive mental health data.
- A **recreated dataset** may increase participant openness/honesty.
- Data **download** is beneficial to researchers without a strong internet connection.
- Data **download** does not offer control over data sharing after the fact.
- Data that is **downloaded** is easy to manipulate.
- There should be data that you can **download** and make it self-destruct.
- A hybrid of these, such as **server** view or a **recreated dataset** if researchers desire download, is needed.

7. What kind of research can people do on the data?

- Maybe **anything** is okay as long as we have exerted control over the other six questions.
- **Anything** is good because we never know what types of research possibilities may be out there.
- Data that is used for **certain types of analysis** or **certain types of projects** would prevent use in marketing or advertising.
- Data that is used for **certain types of analysis** or **certain types of projects** would prevent misuse.
- Data should only be used for **certain types of projects** or **certain types of analysis**, such as mental health or broader health research.

1. Who can access the data?

There was no universal consensus. To summarise the argumentation below, participants largely thought that while it was socially just for **anyone** to access the data, it would be prudent for **people with certain jobs** or **certain skills** to handle the data to ensure informed use.

Common lines of reasoning:

- Allowing **anyone** to access the data is a means of ensuring equality.

[T]his data should be accessible to all because mental health [problems] *is not limited to some specific people* […] *from poor to affluent people, to those who are educated* [and those who aren't] *they to have it* [mental health problems] *and so I feel that anyone should be able to access this data.*
India Session 2, translated to English

- **Anyone** is okay as long as it's for a good reason and/or purpose.

I think when I say "anyone" I am still thinking about people who are working towards specific kinds of goals or like the purpose of usage.
Multinational Session 9

- When **people with certain jobs** or **certain skills** handle the data, they know how to use it properly and/or they won't misuse it.

People with certain jobs and certain skills can access the data. For me that's more acceptable, because in that way, the information can be used in a much better way without being misused.
India Session 7

- Just because people have **certain jobs** or **certain skills** doesn't mean they'll do the right thing.

Just because you're a doctor doesn't mean you can go and use this data for something else.
UK Session 3

- **People from certain places** is discriminatory.

[S]haring or accessing the data, uh, only by the people who – from certain places, I would say that is out of bounds, because it, uh, like, it would be outrightly – outright discriminating in the first place
Multinational Session 1

- **People from certain places** limits the cross-cultural accessibility of research.

Also, I feel that people from certain places shouldn't, like that shouldn't be a barrier, because there could be some cross-cultural studies or some international study that might require the data.
India Session 7

2. *Where is the data hosted?*

This question possibly had the closest to broad convergence of support for **many places** over **one place**.

Common lines of reasoning:

- When data is stored in **many places** it offers us decentralised power and control.

Hosting data in many places also would make sure not one authority is in charge of the data and thus, the control of data is more distributed
Multinational Session 3

- The data should be held in **many places** but not *that* many.

I think there should be at least one backup. So, many places but not too many places because, the more places that have the data, the easier it is for people to access it and steal it.
Multinational Session 9

- The data should be held in **many places**, but everyone should follow GDPR and/or a similar data protection law.

Um, I would say that it would be important for any place where it would be held. Um, any country um, would need to have certain data protection laws in place. So that wherever the data is kept um, it's still protected.
Multinational Session 9

- Data can't be stored in only **one place** because a fire could burn the data centre down.

But then again, if data is stored only in one place, what happens if the computer lab burns down or something?
South Africa Session 3

- Data can't be stored in only **one place** because it could be hacked or lost.

I think one place would be dangerous because if it's at one place, once it's gone it's gone, you can't get it back, but if you have it in different places, we still can have the data again, we can recover it.
UK Session 1

- When data is stored in **one place** it offers us more privacy and control.

If the stuff is extremely sensitive it's better to invest in protection for that one place.
UK Session 6

- When data is stored in **one place** it offers ease of management/organisation.

I guess having it in one place makes it easier to monitor who's accessing it, how it's being passed around.
UK Session 1

3. Who controls the data?

There was no universal consensus. Participants broadly did not want **no one** to control the data, but they debated a range of community-based options. Many participants disliked **community hires manager**, or they wanted to combine **community decides**, **community hires manager**, and **community review panel** in some manner.

Common lines of reasoning:

- **No one** is concerning because you don't know what could be done with the data.

Yeah, I think for me, unacceptable is, no one controls it, because then it's just an absolute free for all and you have no idea where it'll end up.
Multinational Session 6

- **Community decides** would be the best way to fairly represent the entire community.

I think it should just be community, because let's say you've got 10,000 people in this community, you can't represent that via one person, so that rules out the manager idea. And then you can't really get six people only from three countries if you've got 20 in this community. You can't really do that either. So the review panel won't really work either. And people wouldn't be comfortable entrusting it to an algorithm, or some people wouldn't be. So I think community just seems to be the best solution.
UK Session 6

- **Community decides** would be the best ideally, but it would involve too many people, making it chaotic and/or time consuming.

I think it should be a community review panel, mostly because it feels like if the entire community decides, I think that there'll be too many opinions, there could be too many disagreements and agreements and there could be a lot of misunderstanding, maybe.
India Session 7

- **Community decides** could result in a vocal minority having an outsized say.

I think from my side, why I think the community deciding should be unacceptable is because a community can be easily influenced into anything so […] *people who are more clued up about the data should be the one to decide.*
South Africa Session 5

- **Community hires manager** could concentrate power unduly in one person, leading to bias or overwork.

I really don't like the idea of a manager because there are bad eggs everywhere and you don't want to give one person that amount of power.
UK Session 4

- **Community hires manager** would be too burdensome for one person to manage such a big dataset.

I think unacceptable, or probably like something that's probably not possible, is hiring one manager, and it will be too burdensome for one person to take care of the entire data set.
India Session 4

- A **community review panel** would be more representative of the community and/or representative of areas of expertise.

So, I feel like having a community review panel, you know, may also allow uh, room for more diversity and, you know, inclusion of differ – different communities and social groups that could weigh on the decision.
Multinational Session 9

- A **community review panel** may be too bureaucratic.

Um, but also, a disadvantage would be, like, the time, the time aspect. […] *So, it would just take longer for someone to access the data, um, if there's loads of people just trying to decide whether someone should be able to access it.*
Multinational Session 1

- We would like a hybrid of **community decides** + **community hires manager** or **community decides** + **community review panel**.

So the community should decide, primarily, but when it comes to executing those, I guess, there will always be a divide in a community. So I think it should be a combination of community decides and having a manager so that the manager can work according to the guidelines of the communities decisions.
India Session 6

4. What do people have to do before they can access the data?

Most participants wanted a combination of these options. **Ethics training** and **review board approval** were fairly widely supported. **Provide ID** and **sign a contact** were more contentious. The option **pay money** was highly debated.

Common lines of reasoning:

- Requiring researchers to **provide ID** would help us hold people accountable.

So I think the best options will be providing ID, […] *One can one be held accountable if anything goes the other way, wrong way.*
India Session 8

- Requiring researchers to **provide ID** could help us correctly identify people.

I'd identity [identify] *provide ID in acceptable as an identification because we need that for you know, who you are, and for the purpose of identification of this.*
India Session 5

- Requiring researchers to **provide ID** could be discriminatory.

Um, and then also, with providing ID, […] *not everyone has ID as such. Like, certain countries have different documentation. There's undocumented people that want to do research as well, and it shouldn't be, um, uninclusive in that sense, that you have to show ID.*
Multinational Session 1

- A **contract** can be forged/one could deny that one signed it.

Um, I'll go with signing the contract, because lately people be denying everything they've signed on […] *and mostly you can even win the case, because there's no proof that you have signed that thing; and it was you at that time with the document or contract*
South Africa Session 3

- A **contract** could state the purpose for wanting to access the data.

I think…it should be a form where their basic details and their…what purpose they'll use the data for, an explanation of that. That contract should have an explanation that this- for this thing they will use the data and with that move ahead.
India Session 3, translated to English

- A **contract** offers accountability for misuse.

Um, signing contract is really important to me. Um, because people need to be held accountable um, if anything were to happen, if any of the data were compromised.
Multinational Session 9

- Requiring researchers to **pay money** could be discriminatory.

So I think paying money is again, like completely unacceptable for me too, because, again, limit the data to only certain people are probably some big giant people.
India Session 4

- Requiring researchers to **pay money** helps sustain the databank.

In my opinion, even paying money would— should be acceptable. Because for this bank of our data, from this- those fees can be used to manage that bank.
India Session 3, translated to English

- Requiring researchers to **pay money** demonstrates buy-in that protects against misuse.

Um, that it could just facilitate a better quality of research if universities or companies are having to pay for it. There's got to be more of an um, it's more of an incentive to do better research.
Multinational Session 9

- Requiring researchers to **pay money** could be done on a sliding scale.

I think there's a lot of scenario-based things that you need to consider. So, for people who access it, if it's a massive pharmaceutical company, then they should be paying, but if it's a student in a uni who doesn't have much funding, then they shouldn't have to pay as much/at all.
UK Session 3

- Requiring researchers to **pay money** may instil corruption.

I think it could give people ulterior motives, and I think this is bad for me, but I think people could do the worst with it. They can just pay some money and then get that data and then they can just do what they want. So I think that's just a big red flag to me.
UK Session 9

- A **review board** could assist in ascertaining researchers' economic backgrounds for a sliding scale option.

That is, they [review board] *can do a background check before giving approval* [or not]. […] *After that, if the ethics board finds that everything is in order, they will be able to proceed to signing the contract. If its a company, then money can be taken from them. If its not a company, if they are a normal person, who wants to do research, then we can see if we can modify the pay money step for them.*
India Session 2, translated to English

- A **review board** should be utilised for sensitive, contentious, or for-profit projects.

And for studies that are particularly sensitive, I think the additional review board approval would be good.
India Session 6

- Some people might just click through/fast forward an **ethics training**.

[T]here will be a possibility that the people attending it might not be paying attention or you can just fast forward it, especially if it's a video.
Multinational Session 5

- Some professionals already have a code of ethics and do not need an **ethics training**.

If you're part of like, a research institute or organization, you could have fewer like, things. Because if you're already, you know, authorize uh, like a qualified researcher, then you might not have to do as much.
Multinational Session 5

5. Who takes on the cost of managing the data?

This may be the least broadly agreed upon question. This question may also have the most variability by country. **Organisation/institution** was probably the most commonly supported option, but again, this question was not widely agreed upon.

Common lines of reasoning:

- [**People who access it**: lines of reasoning mirrored **pay money** above.]

Yes, I think the people who access it as well as an organization should bear the cost of storing and managing the data. And the pricing for the user should be based on how they want to use the data. For example, if there is profitability involved, then the users should be asked to pay

more. If they are using it, if they are using the data strictly for research purposes, then in that case, only a nominal fee should be charged.
India Session 1

- A **government** is an obvious funder of the databank because the databank serves the welfare of the people.

If the research can help the general public, then the government should be contributing towards that, just like they give money for the NHS
UK Session 3

- A **government** shouldn't fund the databank because that is taxpayers' money—like a backdoor way of making people pay for it.

[I]f it is coming from the government, meaning coming from taxpayers money, the government could possibly in turn um – increase taxes by saying that the money is going to be going towards this, that's why they have to do it.
Multinational Session 7

- The **government** funding the databank would only be used to further their political agenda.

If it's something that could benefit the opposition party. So, say, we have a right-wing government and it's something that supports the left wing, it could impact the more right-wing supporters. It could lead them to not funding it. So that's why it maybe leans more towards 'maybe' than 'acceptable'.
UK Session 7

- Having only one **government** pay for the databank would be unfair, as it is global.

I feel like it's only fair that every institution in any country that's involved in the research should pay for the cost of managing the data because if one government from one country pays for it then it's not really fair on that government.
South Africa Session 8

- Not all **governments** would be able to afford or prioritise funding the databank.

[Y]ou can get certain governments or certain communities within the countries to pay, and they might not want to do it because they have other needs, so they want to focus on healthcare and education and prisons, and whatever. So, it would just – mental health wouldn't be the top priority.
UK Session 2

- If a **private company** funds the databank, they will have an outsized say in what is done with the data.

I think that I would also put private company in 'unacceptable' because I feel like if they're the ones paying for the data to be managed, then they'll feel like they would automatically want to

have some element of control over it, and then having control would mean that if they have a specific agenda.
UK Session 10

- Having a **private company** fund the databank is reasonable if the company is making a product that benefits people with mental illness.

They can make a profit, but if it's ultimately benefiting people, then I'd say that's fine. […] *I also think companies should be able to access it for the purpose of improving their services.*
UK Session 11

- Having a **private company** fund the databank may benefit the company, but it benefits us in that it helps sustain the databank.

One of the advantages of private companies is that if they are spending for the app, then we can help them too. […] *[I]f they also take responsibility for the expenses in our app, then we can create a convenient route for them to get data.*
India Session 2, translated to English

- If a **private company** funds the databank, they may use it to make targeted ads.

I was also going to say, yeah, getting targeted ads based on the stuff that you look at, the last thing you really want is them being able to have access to private data about things like, for example, your mental health, because the last thing you want is something saying, "Oh, are you struggling from depression? Try Jack Daniel's whiskey."
UK Session 11

- If a **private company** funds the databank, they may leak our information.

[I]f a company handles the data, then it could be easily manipulated, and it could be obviously a lot of employees from the company would have access to it. […] *So that's why I wouldn't want the company to have access to the databank and to the information in it.*
India Session 4

- A hybrid of several of these (such as **government** + **organisation/institution** + **private company**) is needed to fund the databank.

I think it should be a combination of government or on an organisation, or institution that is actually collecting the data.
India Session 6

6. How can people see the data?

View it in a server may be the most popular option. More concerns were surfaced about **download** and **recreated dataset**.

Common lines of reasoning:

- A **server** is a secure option that lacks the disadvantages of other options.

View it on a server is acceptable. It's an easier way to protect people's information.

South Africa Session 3

- A **server** makes it challenging to do statistical analyses.

I do believe, for practical reasons, it is better to download the data from the researcher's point of view. Just from personal experience, doing things in the server can be very tedious and very chaotic.
UK Session 8

- A **recreated dataset** may not be granular enough to capture our diversity.

[I]t ignores the individuality kind of. Like if someone has two intersectionalities like they're black and gay and the dataset is recreated and it only takes into account one of them because it averages it out, it sort of ignores the […] *small pockets of people in that dataset.*
UK Session 4

- A **recreated dataset** is needed for its privacy because this is sensitive mental health data.

I do think the recreated dataset, I guess, is a way of just avoiding all risks. And then like it doesn't really matter if someone downloads it or if it gets hacked into I guess, because it's not actually anyone's data. So then you can kind of let people do what they like with it if it's a recreated set.
UK Session 4

- A **recreated dataset** may increase participant openness/honesty.

If I knew that the data was recreated after like I'd submitted my data, I'd be more willing to be honest in the research, if that makes sense. […] *Because if you know it's anonymised a lot and you cannot be traced back, people are more willing to tell the truth kind of.*
UK Session 4

- Data **download** is beneficial to researchers without a strong internet connection.

Netflix also has the download option, um, and I think the best, uh, like the best thing about the feature is that if you don't have internet access […] *[T]here's a lot places all over the world that don't have access to the internet or have, like, weak access to the internet. So, being able to download it is better than being able to view it in a server.*
Multinational Session 1

- Data **download** does not offer control over data sharing after the fact.

If you can download it, then that's a problem because you will have it on your computer; and we are not sure what you're going to do with the information that you got; and it's not as private as it was going to be if you could only view it in a server.
South Africa Session 8

- Data that is **downloaded** is easy to manipulate.

I also agree with data being downloaded is unacceptable because people can easily tamper with the data.
South Africa Session 8

- There should be data that you can **download** and make it self-destruct.

I think I said last time as well that we could have like a temporary type of download. So, there's – sometimes you have this thing where you can download something and it's like a disappearing download. So, it will run out after a certain amount of time.
Multinational Session 7

- A hybrid of these, such as **server** view or a **recreated dataset** if researchers desire download, is needed.

ACTUALLY what if we had recreated data as downloadable and then real data to be viewed on a server!!
UK Session 6

7. What kind of research can people do on the data?

Anything was not widely supported. Most participants wanted **certain types of analysis** or **certain types of projects**.

Common lines of reasoning:

- Maybe **anything** is okay as long as we have exerted control over the other six questions.

Uh, so I feel it should be like, I – I would put anything in acceptable because uh, after so many filters that we will have put, uh, going through the board approval and then signing a contract uh, I feel putting it down to certain types of analysis and certain types of projects should not be a necessity.
Multinational Session 3

- **Anything** is good because we never know what types of research possibilities may be out there.

Many people come up with new things regarding like, anything like they can come with new things besides being maybe analyzing on certain product project or analysis. They can come up with bring new things regarding the data. So anything is acceptable for me
South Africa Session 6

- Data that is used for **certain types of analysis** or **certain types of projects** would prevent use in marketing or advertising.

Obviously, I don't know how likely it is, depending on the data collected, but I think people who would have very obvious, I guess, interests with advertising and wanting to figure out how to target a certain teenaged demographic who has mental illnesses, and stuff like that, I wouldn't really want the data being used for that.
UK Session 1

- Data that is used for **certain types of analysis** or **certain types of projects** would prevent misuse.

I think that anything could … I think if anyone had access to it and it could be used for anything, it's not necessarily necessary for all projects and analysis, but, also, some people could misuse the data and try and get a conclusion from it which would benefit their business.
UK Session 7

- Data should only be used for **certain types of projects** or **certain types of analysis**, such as mental health or broader health research.

For that, I would say it should be for mental health projects, and analysis, because I feel that that's the place where the data collection is the most useful, according to my knowledge, and my it's my opinion over it. And I feel that is the place where the data is secured, also, and as it's in the right people's hands, and they have complete decency of protecting the data.
India Session 5
