# Supplementary material for: Young people’s data governance preferences for their mental health data: MindKind Study findings from India, South Africa, and the United Kingdom
Source: PLoS One. 2023 Apr 19;18(4):e0279857. doi: 10.1371/journal.pone.0279857 (PMC10115253; doi:10.1371/journal.pone.0279857)
Supplement: Attachment — Submitted filename: Response to Reviewers.docx [file pone.0279857.s002.docx]

Response to Reviewers

Young people’s data governance preferences for their mental health data: MindKind Study findings from India, South Africa, and the United Kingdom

We appreciate the reviewers’ supportive comments as well as the thoughtful concerns raised. We have addressed each one below. The reviewers’ comments are quoted in **black** and our responses are in **red**.

**Reviewer 1**

When the authors state "young participants", they include participants aged 16-24 in the UK and 18-24 in the other regions. Why didn't you select the same age interval between countries? I do not see the benefits of including differential age groups between sites, please explain.

This study included participants from the age of consent (18 in India and South Africa, and 16 in the UK) to 24 years of age. We recognize that this introduced some differences across the countries examined, however we thought it important to include the youngest participants who were able to provide their own consent without the input of a parent/guardian. For the quantitative study, we did examine the effect of age as noted in the text. Age was not significant in the preferences related to ‘how researchers access the data’ nor in the analysis of participant acceptability. We did notice a modest effect (p-val =0.05) for the choice of ‘who controls access to the data’ with younger participants being more likely to choose professional review panel.

For the qualitative study, we included participants of the same age ranges to best produce results that corresponded to the quantitative study. We separated groups by younger (16-20) and older (21-24) participants to minimize power dynamics between participants, which we detailed in the Materials and methods as follows: “Across all sessions, we sought stratification of younger (16-20) and older (21-24) participants…”

We have added the following text to the discussion:
“We chose to include participants ranging from the age of consent (i.e. those legally allowed to make decisions about study participation without the need for a parent/guardian) to the age of 24, in order to understand the choices that young people would make for themselves. This resulted in differences, by country, in the ages included (18-24 for India and South Africa; 16-24 for UK). However, we largely found no effect of age in our analysis, except for a minor increase in those choosing a professional review panel to control access to their data. In the qualitative study, we stratified groups by age in order to minimize power dynamics between participants.”

Please explain what is a "diverse youth" based on some variables.

Thank you for bringing this to our attention. We wrote “PYAs sought to ensure members represented diverse youth from their countries based on geographic region, race/ethnicity, gender, language, and lived experience of mental health concerns.” We recognize that this language was unclear as to how the seeking of diversity was operationalized in our Young People's Advisory Groups (YPAGs). We have modified this language as follows: “Each YPAG was composed of 12-16 young people, and PYAs sought to ensure maximal heterogeneity within the groups with respect to geographic region, race/ethnicity, gender, languages spoken, and lived experience of mental health concerns. Study teams also worked with community partner organizations to ensure the representation of YPAG members from marginalized social groups.”

Why the discrepancy on the % of participants allocated by social media advertisements between sites? Elaborate the possible impacts on the results.

Each site employed a variety of different approaches to recruitment, however unique attributes of each country likely affected which approaches were successful. For example, the high cost of data, load shedding, etc in South Africa likely contributed to online recruitment being less successful here. We have a forthcoming publication delving into this topic so have not discussed it in-depth in this manuscript. We have added the following note in the limitations section:
“We also noted that a larger portion of participants were recruited through offline strategies.”

As you state in the discussion section, Android users represent only part of the population, possible selection bias?

Yes, we have noted this in the discussion “While Android phones have good market share in the UK [34], they tend to be substantially less popular among younger users [35], **so quantitative results may not be generalizable to the rest of the UK population**.” We have added “In contrast, Android phones are highly represented in India and South Africa [36, 37], though the Android requirement is a potential limitation here as well.” to make clear that the Android requirement affected representativeness in all three countries.

**Reviewer 2**

I ask you to clarify in the article the fact of selecting participants with ages in different ranges. Would that imply a bias? What did you do to mitigate this limitation?

This study included participants from the age of consent (18 in India and South Africa, and 16 in the UK) to 24 years of age. We recognize that this introduced some differences across the countries examined, however we thought it important to include the youngest participants who were able to provide their own consent without the input of a parent/guardian. For the quantitative study, we did examine the effect of age as noted in the text. Age was not significant in the preferences related to ‘how researchers access the data’ nor in the analysis of participant acceptability. We did notice a modest effect (p-val =0.05) for the choice of ‘who controls access to the data’ with younger participants being more likely to choose professional review panel.

For the qualitative study, we included participants of the same age ranges to best produce results that corresponded to the quantitative study. We separated groups by younger (16-20) and older (21-24) participants to minimize power dynamics between participants, which we detailed in the Materials and methods as follows: “Across all sessions, we sought stratification of younger (16-20) and older (21-24) participants…”

We have added the following text to the discussion:
“We chose to include participants ranging from the age of consent (i.e. those legally allowed to make decisions about study participation without the need for a parent/guardian) to the age of 24, in order to understand the choices that young people would make for themselves. This resulted in differences, by country, in the ages included (18-24 for India and South Africa; 16-24 for UK). However, we largely found no effect of age in our analysis, except for a minor increase in those choosing a professional review panel to control access to their data. In the qualitative study, we stratified groups by age in order to minimize power dynamics between participants.”
